# The Effect of the Nasal Airflow Reducer on Parasympathetic Activity in Adults: A Pilot and Exploratory Study

**DOI:** 10.3390/medicina61101772

**Published:** 2025-10-01

**Authors:** Yen-Chang Lin, Jui-Kun Chiang, Hsueh-Hsin Kao, Tzu-Hao Lin, Tzu-Ying Hung, Yee-Hsin Kao

**Affiliations:** 1 Nature Dental Clinic, Puli Township, Nantou 545, Taiwan; drlin@alliswell.tw; 2 Department of Family Medicine, Dalin Tzu Chi Hospital, Buddhist Tzu Chi Medical Foundation, Chiayi 622, Taiwan; roma@tzuchi.com.tw; 3 Department of Radiation Oncology, Taichung Veterans General Hospital, Taichung 407, Taiwan; kaogrady8176@gmail.com; 4 Department of Biomedical Engineering, Columbia University, 116th and Broadway, New York, NY 10027, USA; tl3460@columbia.edu; 5 Department of Family Medicine, Tainan Municipal Hospital (Managed by Show Chwan Medical Care Corporation), Tainan 701, Taiwan

**Keywords:** heart rate variability (HRV), analysis, parasympathetic, body mass index (BMI)

## Abstract

*Background and Objectives*: Boosting parasympathetic activity may enhance both physical and mental functions. In this study, we introduced the Lin Nasal Airflow Reducer (L.NAR), a silicone device designed to reduce nasal airflow. This pilot and exploratory study aimed to investigate the effect of L.NAR on parasympathetic activity in adults. *Materials and Methods*: The test protocol consisted of two 16 min ECG sessions. In the first session, participants did not wear the L.NAR for the initial 8 min (Test 1) but wore it for the remaining 8 min (Test 2). Following a 30 min rest, the second session reversed the sequence, with participants wearing the L.NAR for the first 8 min (Test 3) and removing it for the final 8 min (Test 4). Time- and frequency-domain analyses and non-linear analyses were used to assess heart rate variability (HRV) for every 300 s moving by 10 s. Repeated measurement ANOVA was conducted to compare the means across the four tests. *Results*: A total of 49 participants were enrolled in the analysis, with a mean age of 40.3 ± 10.7 years. Male participants had a higher body mass index (BMI) than female participants (24.0 ± 3.3 vs. 21.3 ± 2.9 kg/m^2^, *p* = 0.014). Participants in Test 3 and Test 4 had significantly lower heart rate values than those in Test 1. Participants wearing the L.NAR (Test 2 and Test 3) had significantly higher RMSSD values compared to those not using the L.NAR. Among the participants, 33 (67.3%) who wore the L.NAR showed significantly higher RMSSD levels compared to their pre-L.NAR levels during the first practice. This improvement was achieved after an average of 2.5 ± 2.9 sessions. *Conclusions*: In this study, we introduced a novel approach using the L.NAR to increase RMSSD, a key indicator of parasympathetic activity.

## 1. Introduction

The parasympathetic system decreases heart rate to conserve energy at rest. It enhances salivary secretion to aid in swallowing, stimulates gastric motility and secretion to process ingested food, and promotes intestinal motility and secretion for nutrient absorption. It also stimulates both exocrine and endocrine secretions from the pancreas: exocrine enzymes aid in intestinal food breakdown, and endocrine insulin facilitates nutrient storage in tissues. Additionally, the system induces urinary bladder contraction for urination and adjusts the eye for near vision by contracting the pupil (miosis) and adapting the lens [1]. However, a previous study reported that negative affect, which encompasses both mood and emotions, may be associated with a reduction in parasympathetic activity [2]. There are many methods to increase parasympathetic activity, such as regular exercise, deep breathing and walking, and others.

Furthermore, moderate pressure massage has been shown to enhance parasympathetic nervous system response during the first half of a session [3]. Several pharmacologic interventions for congestive heart failure, such as angiotensin-converting enzyme inhibitors, angiotensin receptor blockers, beta-adrenergic antagonists, digoxin, and vasodilators, may positively influence the parasympathetic nervous system, potentially improving symptoms and prognosis [4]. By stimulating the vagal afferent pathway to the frontal cortical areas and increasing baroreflex sensitivity, heart rate variability biofeedback (HRVB) is used to treat a variety of conditions, including asthma [5], functional abdominal pain [6], hypertension [7], posttraumatic stress disorder [8], depression [9], and performance enhancement, among others. Additionally, a significant increase in the root mean square of successive heart beat interval differences (RMSSD) was observed following a low-fat diet in healthy premenopausal women [10]. Another study reported that ultrasound-guided percutaneous needle electrolysis, which combines needle puncture with electric current, resulted in a measurable increase in parasympathetic activity [11].

Previous studies have reported that incorporating hypoxia into training induces higher metabolic stress, particularly in skeletal muscle, by reducing intramuscular oxygen partial pressure [12]. Recent data also indicate that repeated sprint training in hypoxia improves repeated sprint ability in team-sport players [13]. In the current study, we introduced a novel, patented nasal appliance known as the Lin Nasal Airflow Reducer (L.NAR), which effectively induces near-complete nasal obstruction to reduce breathing volume. The rationale behind the innovation of the L.NAR is to create a hypoxic situation to increase parasympathetic activity. This might involve reducing airflow and consequently decreasing breathing volume to generate inhibitory signals and hyperpolarizing currents within neural and non-neural tissues by mechanically stretching them during inhalation and breath retention. Then, inhibitory impulses, along with hyperpolarization currents, likely initiate the synchronization of neural elements in the central nervous system, peripheral nervous system, and surrounding tissues. This process ultimately causes a shift in the autonomic balance towards parasympathetic dominance [14]. The well-documented effects of hypoxic training include increased mitochondrial density, heightened oxidative enzyme activity, enhanced capillary thickness, and a general shift from the utilization of fat and muscle glycogen to the combustion of blood glucose [12,15,16]. Another possible rationale is the reduction in respiratory rate. A previous systematic review demonstrated that voluntary slow breathing increases vagally mediated heart rate variability (HRV), supporting the proposed mechanism that slow breathing stimulates vagal nerve activity [17]. The relationship between parasympathetic activity and hypoxic conditions is significant and warrants comprehensive investigation. Another potential mechanism of the L.NAR device is that it may induce slow breathing when worn, similar to pranayamic breathing [18], which is known to enhance parasympathetic activity. The objective of the present pilot and exploratory study was to introduce a novel method using the L.NAR device to assess its impact on parasympathetic activity in adults, measured with a wearable Polar H10 sensor.

## 2. Materials and Methods

### 2.1. Study Design

This observational study was conducted to explore the impact of the L.NAR on HRV in adults. The study protocol was reviewed and approved by the institutional review board of the Research Ethics Committee of the Buddhist Dalin Tzu Chi Hospital, Chiayi, Taiwan (No. B11204016).

This study enrolled 54 adults who voluntarily attended a dental clinic between 1 January 2024 and 30 September 2024. The inclusion criteria included being 20 years of age or older and willing to participate in the study. The exclusion criteria included participants with hypertension, coronary artery disease, arrhythmias, cerebrovascular accidents, diabetes, a history of smoking, alcohol consumption, or stimulant drink use, as well as those who were fearful of using the L.NAR device. All procedures were conducted in accordance with the ethical standards of the responsible institutional and national committees on human experimentation, as well as with the 1975 Helsinki Declaration, as revised in 2008. Participation was voluntary, and written informed consent was obtained from all participants prior to enrollment. All participants wore the L.NAR and underwent HRV testing.

### 2.2. Introduction of L.NAR and Testing Procedure

**Figure 1:**
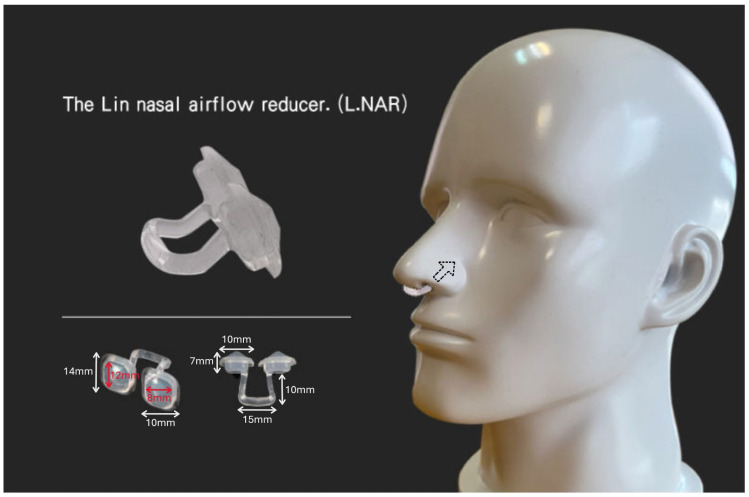
The structure of the Lin Nasal Airflow Reducer (L.NAR).

We are introducing a plastic (silicon) device designed as a nasal airflow reducer: the Lin Nasal Airflow Reducer (L.NAR), which effectively induces nearly complete nasal obstruction to reduce breathing volume. L.NAR is constructed from medical-grade silicon and consists of two components: two rhomboid-shaped pieces placed in the nasal orifices and a connector linking these two rhomboid pieces. The weight of the L.NAR is approximately 2 g (Figure 1). The patent number for the L.NAR is I846581 in Taiwan [19].

**Figure 2:**
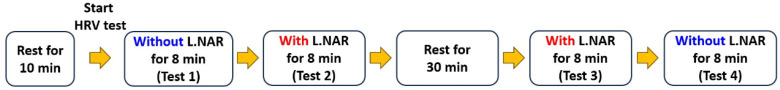
Study flow chart.

To reduce the bias of time effects, we designed the following flowchart. The test procedure was as follows: Participants rested for 10 min before the practice session. A 16 min electrocardiography (ECG) session was conducted, during which participants did not use the L.NAR for the first 8 min (Test 1) but wore it for the following 8 min (Test 2). After a 30 min rest, another 16 min ECG session was performed, this time with participants wearing the L.NAR for the first 8 min (Test 3) and removing it for the remaining 8 min (Test 4) (Figure 2). Participants could repeat the test procedure on different days throughout the study period if they wished to obtain more reliable experimental results.

The electrodes of a Polar H10 device (Polar Electro Oy, Kempele, Finland) were dampened with room-temperature water and placed on the xiphoid process of the sternum. The chest strap was securely fastened around the participant’s chest, just below the chest muscles [20]. The Polar H10 ECG unit records electrical heart signals and has been validated against a three-lead ECG, which is considered the gold standard in this field [21]. RR intervals were recorded via Bluetooth using the Polar Sensor Logger application (version: 3.3.2, Polar Electro Oy, Kempele, Finland) to obtain raw data for analysis and processing [22]. We instructed participants to breathe through their nose as usual and avoid mouth breathing throughout the entire test.

### 2.3. HRV Measurement

Evaluating heart rate variability (HRV) provides a non-invasive, reliable, and pain-free method for assessing autonomic nervous system activity. The root mean square of successive heart beat interval differences (RMSSD, milliseconds) was utilized as an indicator of parasympathetic activity. The LF/HF ratio, defined as the absolute power of the low-frequency (LF) band (0.040–0.150 Hz) divided by the absolute power of the high-frequency (HF) band (0.150–0.400 Hz), was used as a marker of sympathetic activity.

In the current study, we utilized the low-frequency power to high-frequency power ratio (LF/HF ratio), a frequency-domain measurement index, as a surrogate for sympathetic activity, in accordance with the methodologies described in previous studies by AlQatari et al. [23] and Kobayashi et al. [24]. Additionally, we used RMSSD and lnHF (the natural logarithm of HF) as surrogate measures of parasympathetic activity, with RMSSD evaluated using the time-domain method [25,26] and lnHF using the frequency-domain method [27].

### 2.4. Outcome Measures

The objective of this pilot and exploratory study was to investigate the effects of the L.NAR on sympathetic and parasympathetic activity across four tests in adults.

### 2.5. Statistical Analysis

Continuous data were presented as mean ± standard deviation (SD) and compared using a *t*-test or Wilcoxon rank-sum test as indicated. Electrocardiogram data were downloaded, and all analyses were conducted using R software (version 4.4.1, R Foundation for Statistical Computing, Vienna, Austria). Data files were visually inspected for artifacts, with corrections applied either manually or through the software as needed.

HRV analysis was performed using a 300 s window with a 10 s shift. The HRV results from these time series data were selected for Tests 1 through 4. Correlations between HRV parameters were analyzed, and repeated measurement ANOVA for self-crossover tests was used to compare these four tests. If the assumption of sphericity was violated according to Mauchly’s Test, the Greenhouse–Geisser correction was applied. Post hoc tests were conducted using the Bonferroni correction, with results presented as mean differences and adjusted *p*-values. All statistical analyses were two-sided, with statistical significance set at *p* = 0.05. We used the **pwr** package in R software. With the parameters set as follows: Cohen’s *f* = 0.28 (medium effect size, approximately 0.25), *k* = 4, *n* = 40, and significance level = 0.05, the resulting power was 84.78%. We therefore selected *n* = 40, and for safety, determined that *n* = 49 would be satisfactory.

## 3. Results

**Figure 3:**
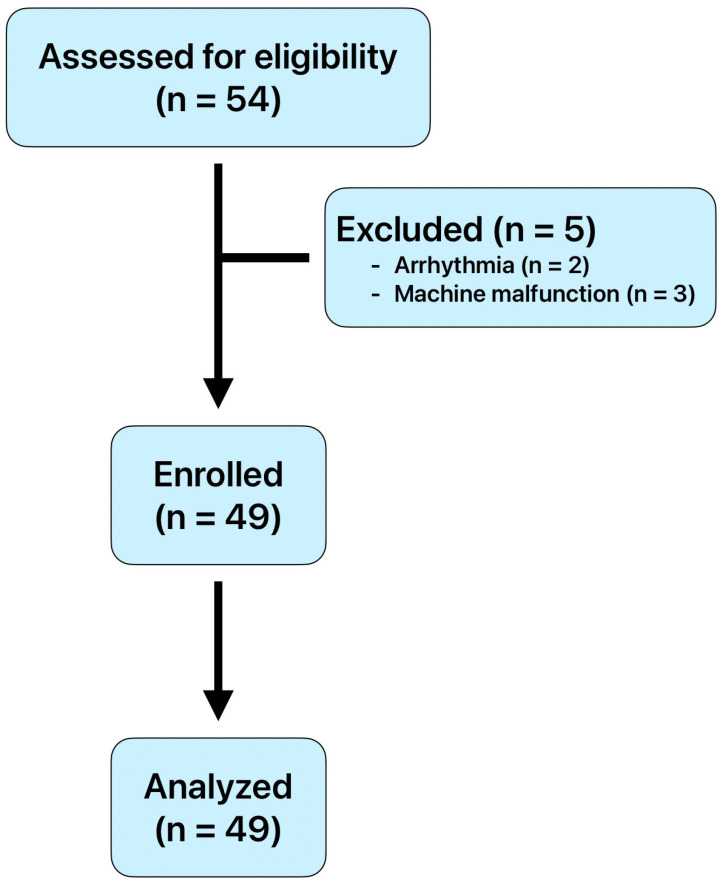
Flow diagram illustrating the recruitment of participants.

In this study, we recruited 54 participants consecutively and initially collected data from all of them. However, we excluded five participants: two due to arrhythmia, and three due to machine malfunctions. Ultimately, 49 participants (29 female and 20 male) were included in the final analysis. A flow diagram illustrating the recruitment of participants is shown in Figure 3. The mean age of participants was 40.3 ± 10.7 years, and the mean body mass index (BMI) was 22.7 ± 3.4 kg/m^2^. Male participants had a higher BMI than females (24.0 ± 3.3 kg/m^2^ vs. 21.3 ± 2.9 kg/m^2^, *p* = 0.014), and there was no significant difference in age between genders (*p* = 0.086) (Table 1). Two participants had a history of hypertension, and two had a history of stroke among each gender.

In this study, we examined the parameters LF, HF, lnHF, LF/HF ratio, and RMSSD, and we found strong correlations between RMSSD and HF (*r* = 0.756, *p* < 0.001), and RMSSD and lnHF (*r* = 0.788, *p* < 0.001) (Table 2). We proposed using RMSSD as a surrogate for parasympathetic activity, and the LF/HF ratio as a surrogate for sympathetic activity.

**Figure 4:**
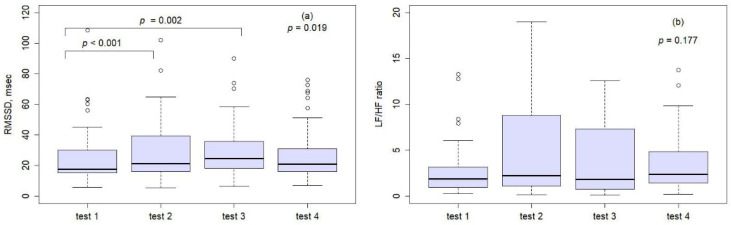
The bar plot for RMSSD (**a**) and LF/HF ratio (**b**) across the four tests.

Among the 49 participants, 33 (67.3%) who wore the L.NAR showed an increase in RMSSD during the first session. On average, participants completed 2.5 ± 2.9 practice sessions, ranging from 1 to 14 sessions. There were significant differences in heart rates among the four tests (*p* = 0.001; Table 3). Post hoc tests with Bonferroni correction revealed that heart rates in Test 1 were significantly higher than those in Test 3 and Test 4, with mean differences of 3.94 ± 6.71 (*p* = 0.001) and 2.83 ± 6.86 (*p* = 0.035), respectively (Table 4). For the RMSSD parameter, significant differences were also observed among the four tests (*p* = 0.019; Table 3). Compared to Test 1, RMSSD significantly increased in both Test 2 (mean difference: –3.91 ± 6.23, *p* < 0.001) and Test 3 (mean difference: –4.43 ± 7.90, *p* = 0.002) (Figure 4a; Table 4). In contrast, no statistically significant differences were found among the four tests for the LF/HF ratio (*p* = 0.177; Figure 4b; Table 3).

## 4. Discussion

In this study, we introduced a novel approach using the L.NAR to enhance parasympathetic activity. Our findings showed that participants reached the goal of increasing RMSSD, a marker of parasympathetic activity, after an average of 2.5 ± 2.9 sessions. Notably, thirty-three participants (67.3%) achieved this goal during their first session while wearing the L.NAR. Although there are many methods to increase parasympathetic activity, such as voluntary slow breathing exercises, wearing the L.NAR offers an alternative approach that does not require patients to consciously focus on their breathing.

To minimize variability in session intervals and testing conditions, we standardized the procedure for participants wearing the L.NAR. All sessions were conducted in a single, quiet, air-conditioned room, restricted to the 2:00–4:00 p.m. time window, and fixed at a measurement duration of 8 min to ensure consistency. Participants demonstrated strong adherence to wearing the L.NAR during the test procedure and generally showed good tolerance throughout the study period. Reported adverse effects were minimal and included mild nasal or facial discomfort, transient sensations of breathlessness, and occasional urges to take deep breaths. These symptoms were subjective, temporary, and did not interfere with the continuation of the test. No serious adverse events occurred. Two participants were withdrawn from the study due to pre-existing arrhythmia. Overall, the device was well tolerated, with participants able to complete the prescribed sessions without interruption, and no participant required discontinuation solely because of intolerance to the L.NAR.

Heart rate variability biofeedback (HRVB) has gained considerable popularity in recent years. A previous study found that HRVB enhances the coordination of emotional networks in the brain by increasing functional connectivity between the left amygdala and the medial prefrontal cortex, as well as improving connectivity within emotion-related resting-state networks during rest [28]. In the study by Nashiro et al., participants followed specific guidelines, practicing for 20 min per day during the first week, 30 min per day in the second week, and 40 min per day for the remaining weeks, with a total of six weeks of training required to achieve the desired effect [28]. Another previous study hypothesized that the central benefits of deep and slow-paced breathing, as well as HRVB, originate in the limbic areas, which may be stimulated by increased oscillations in cerebral blood flow and enhanced oxygen delivery. These mechanisms may, in turn, contribute to improvements in pain regulation, emotional awareness, cognitive function, and stress management [29]. In addition, another study reported that HRV biofeedback consistently induces acute improvements during practice; however, the presence of short-term and long-term carry-over effects remains uncertain [30]. In the current study, participants achieved enhanced parasympathetic activity after an average of 2.5 ± 2.9 sessions. However, the interval time between sessions was not recorded, which may be a limitation of this study. This innovative aid is designed to rapidly stimulate parasympathetic activity, potentially through changes in breathing patterns. The L.NAR appliance is designed to require mouth closure and to encourage nasal breathing. To fully experience the benefits—such as increased parasympathetic activity—participants may need time to adjust to this new breathing pattern. Further research is needed to explore the long-term effects and the influence of usage duration on parasympathetic enhancement with the L.NAR.

Two possible rationales for the increased parasympathetic activity observed while wearing the L.NAR are the effects of pranayamic breathing and a reduced respiratory rate. By having participants wear the L.NAR, we aimed to simulate a condition similar to pranayamic breathing, which is known to enhance parasympathetic activation [14]. In contrast to slow pranayamic breathing, nostril breathing—whether through the right nostril, left nostril, or both nostrils—has been shown to increase baseline oxygen consumption, indicative of sympathetic discharge of the adrenal medulla [31]. In another study using breathing exercises mimicking pranayama, slow breathing over a period of three months was shown to improve autonomic function, while fast breathing did not affect the autonomic nervous system [32]. The definite respiratory volume per minute was not measured while participants used L.NAR, which is a limitation of the current study and warrants further investigation. Another rationale is the slowing of the respiratory rate. A previous systematic review demonstrated an increase in vagally mediated heart rate variability (HRV), supporting the proposed mechanism by which voluntary slow breathing stimulates the vagus nerve. As such, voluntary slow breathing exercises may be recommended as a low-tech, low-cost approach for preventive and adjunctive treatment, with minimal anticipated adverse effects [18]. However, a limitation of the current study is that respiratory rate was not analyzed, either with or without the use of the L.NAR. Further studies are warranted to objectively measure respiratory rates during L.NAR use and at rest, in order to clarify whether the observed effects are indeed associated with a decrease in respiratory rate. In pranayama, individuals autonomously regulate breathing rate and duration and usually remain comfortable. Conversely, L.NAR imposes a nasal airflow constraint that may initially feel non-physiological, modifying both breathing rate and tolerability. Despite voluntary enrollment, some participants reported transient disruptions of breathing rhythm, sometimes accompanied by affective (limbic) distress; intermittent instructor guidance was required. We initially evaluated different durations (30, 20, 10, 8, and 5 min) and found that HRV analysis stabilized at a minimum of 8 min; therefore, this duration was adopted. An 8 min period was considered clinically relevant, as it provided sufficient time to ensure physiological stability while maintaining practical feasibility. However, this study did not assess how long the increase in parasympathetic activity persisted after device use. Future studies are needed to determine both the duration and clinical significance of this effect. In addition, the optimal daily duration and frequency of the maneuvers required for clinical relevance were not evaluated. Further research is warranted to establish a clinically meaningful dose–response relationship.

While the electrocardiogram (ECG) is the gold standard for quantifying RR intervals and assessing HRV, it can be costly and complex, potentially limiting compliance [33]. Portable devices offer a simpler, more practical alternative for field measurements. According to a systematic review, HRV measurements from portable devices show a small but acceptable absolute error compared to ECG, making them a viable option for improving compliance in HRV monitoring [34]. In the current study, we used a Polar H10 sensor chest strap device and “Polar Sensor Logger” app to assess the HRV. A previous study reported that the Polar H10 is the most accurate wearable device [22].

In the current study, we found that the LF/HF ratio, a marker of sympathetic activity, did not show a statistically significant difference across the four tests. One possible explanation is that wearing the L.NAR does not elicit a stimulating response.

The current study has several limitations that should be acknowledged. First, the sample size was relatively small, with only 49 participants (29 females and 20 males) included in the analysis. Second, because this was an observational study, a double-blind design was not feasible given the nature of the intervention. Instead, a repeated-measures, self-crossover design was adopted to ensure the reliability of the results when evaluating the effects of the L.NAR. Third, the L.NAR has only been registered in Taiwan. Expanding sales to other countries would broaden its applicability and help verify its effectiveness in enhancing parasympathetic activity across different ethnic groups. Fourth, HRV measurements were taken during only two practice sessions, each lasting 16 min, rather than through continuous 24 h monitoring. Therefore, further evaluation is necessary to determine whether the increase in parasympathetic activity persists after using the L.NAR. Fifth, this study did not analyze respiratory frequency, either with or without the use of the L.NAR. Sixth, additional measures of autonomic activity, such as pupillometry, muscle sympathetic nerve activity recordings, Valsalva responses, norepinephrine, and electromyography, should be incorporated alongside HRV for a more accurate assessment of sympathetic and parasympathetic activity. Seventh, HRV evaluation can be influenced by various factors, including temperature, humidity, stimulant use, as well as the health status and lifestyle of participants. Although participants self-reported abstaining from stimulants such as alcohol, coffee, and tea, adherence to these guidelines was not strictly monitored, which represents a limitation of this study. Additionally, lifestyle factors such as regular physical activity were not assessed. Eighth, the study did not adjust for chronic nasal obstruction (e.g., deviated septum, turbinate hypertrophy, narrow palate), potentially increasing variability in the observed effects during a single session. Even so, assessing within-individual temporal changes minimizes underestimation. Last, the duration for which participants can tolerate the L.NAR also needs to be investigated. However, introducing the L.NAR provides a non-invasive and effective method to increase parasympathetic activity.

## 5. Conclusions

In the current study, we proposed a novel method: using the L.NAR to increase RMSSD, a key indicator of parasympathetic activity. Among 49 participants, 33 (67.3%) who wore the L.NAR achieved the goal in the first session, and all could achieve satisfactory results after practice.

## 6. Patents

The patent number for the Lin Nasal Airflow Reducer (L.NAR) is I846581 in Taiwan.

## Figures and Tables

**Table 1:** Demographics and characteristics of the participants enrolled.

Variable	Total	Female	Male	*p*
Gender (female/male)	49	29 (59.2%)	20 (40.8%)	-
Age, years	40.26 ± 10.68	37.63 ± 8.09	43.72 ± 12.81	0.086
BMI, kg/m^2^	22.69 ± 3.37	21.34 ± 2.91	24.04 ± 3.32	0.014

Abbreviations: BMI, body mass index.

**Table 2:** Correlations between variables of autonomic nervous activity measurements.

Variables	LF	HF	lnHF	LF/HF, Mean	RMSSD, Msec
Heart rate, 1/min	−0.223 (0.002)	−0.412 (<0.001)	−0.514 (<0.001)	0.062 (0.389)	−0.567 (<0.001)
LF		0.064 (0.375)	0.134 (0.061)	0.514 (<0.001)	0.268 (<0.001)
HF			0.796 (<0.001)	−0.218 (0.002)	0.756 (<0.001)
lnHF				−0.336 (<0.001)	0.788 (<0.001)
LF/HF, mean					−0.109 (0.127)

Abbreviations: LF/HF ratio, the ratio of the absolute power of the low-frequency band (0.04–0.15 Hz) to the absolute power of the high-frequency band (0.015–0.4 Hz); lnHF, the natural logarithm of HF; RMSSD, the root mean square of successive differences between normal heartbeats.

**Table 3:** Comparison of autonomic nervous activity measurement variables across the four tests.

Variables	Test 1	Test 2	Test 3	Test 4	Df	F	ges	Mauchly’s Test *p* Value	ANOVA	G.G. Correction *p* Value
Heart rate, 1/min	81.7 ± 9.9	79.9 ± 9.6	77.79 ± 8.62	78.89 ± 7.99	144	7.10	0.03	<0.001	-	0.001
LF	166.6 ± 202.0	324.2 ± 464.9	329.7 ± 475.6	195.4 ± 216.7	144	7.09	0.04	<0.001	-	0.002
lnHF	3.8 ± 1.2	4.1 ± 1.4	4.4 ± 1.2	4.0 ± 1.2	144	7.23	0.03	0.229	<0.001	
LF/HF ratio	4.3 ± 7.2	8.5 ± 18.9	7.7 ± 18.0	5.1 ± 10.6	144	1.85	0.01	<0.001	-	0.177
RMSSD, msec	25.1 ± 19.0	29.0 ± 19.8	29.5 ± 17.8	27.8 ± 18.4	144	4.24	0.01	<0.001	-	0.019

In Test 1, participants did not use the L.NAR for the first 8 min, then wore it for the remaining 8 min in Test 2. After a 30 min rest, another 16 min ECG session was conducted, during which participants wore the L.NAR for the first 8 min in Test 3 and removed it for the remaining 8 min in Test 4. Abbreviations: Df, degree of freedom; ges, generalized eta squared; LF/HF ratio, the ratio of the absolute power of the low-frequency band (0.04–0.15 Hz) to the absolute power of the high-frequency band (0.015–0.4 Hz); lnHF, the natural logarithm of HF; RMSSD, the root mean square of successive differences between normal heartbeats.

**Table 4:** The post hoc tests utilized the Bonferroni correction, with results presented as mean differences (adjusted *p*-values).

Variables	Mean	Standard Deviation	*p*
Heart rate, 1/min			
Test 1 vs. test 2	1.87	5.68	0.153
Test 1 vs. test 3	3.94	6.71	0.001
Test 2 vs. test 3	2.07	6.36	0.165
Test 1 vs. test 4	2.83	6.86	0.035
Test 2 vs. test 4	0.96	7.35	1.000
Test 3 vs. test 4	−1.11	3.46	0.176
LF/HF ratio			
Test 1 vs. test 2	−4.17	15.00	0.350
Test 1 vs. test 3	−3.35	13.46	0.530
Test 2 vs. test 3	0.82	5.16	1.000
Test 1 vs. test 4	−0.78	8.94	1.000
Test 2 vs. test 4	3.39	20.34	1.000
Test 3 vs. test 4	2.57	18.56	1.000
RMSSD, msec			
Test 1 vs. test 2	−3.91	6.23	<0.001
Test 1 vs. test 3	−4.43	7.90	0.002
Test 2 vs. test 3	−0.51	9.79	1.000
Test 1 vs. test 4	−2.72	11.06	0.549
Test 2 vs. test 4	1.19	12.59	1.000
Test 3 vs. test 4	1.71	8.06	0.869

Abbreviations: LF/HF ratio, the ratio of the absolute power of the low-frequency band (0.04–0.15 Hz) to the absolute power of the high-frequency band (0.015–0.4 Hz); RMSSD, the root mean square of successive differences between normal heartbeats.

## Data Availability

All the associated data not provided within the paper are available on reasonable request from Yee-Hsin Kao.

## Abbreviations

The following abbreviations are used in this manuscript:

L.NAR	Lin Nasal Airflow Reducer
HRV	Heart Rate Variability
BMI	Body Mass Index
RMSSD	Root Mean Square of Successive Heart Beat Interval Differences
HRVB	Heart rate variability biofeedback
ECG	Electrocardiography
LF	Low-Frequency
HF	High-Frequency
LF/HF ratio	Low-Frequency Power to High-Frequency Power Ratio
lnHF	Natural Logarithm of HF
SD	Standard Deviation
Df	Degree of Freedom
ges	Generalized Eta Squared
